# Feasibility study of adjuvant chemotherapy with S-1 after curative esophagectomy following neoadjuvant chemotherapy for esophageal cancer

**DOI:** 10.1186/s12885-022-09827-3

**Published:** 2022-06-30

**Authors:** Noriyuki Hirahara, Takeshi Matsubara, Shunsuke Kaji, Hikota Hayashi, Koki Kawakami, Yohei Sasaki, Satoshi Takao, Natsuko Takao, Ryoji Hyakudomi, Tetsu Yamamoto, Yoshitsugu Tajima

**Affiliations:** 1 Department of Digestive and General Surgery, Faculty of Medicine, Shimane University, 89-1 Enya-cho, Izumo, Shimane 693-8501 Japan; 2 Department of Surgery, Matsue Red Cross Hospital, Horo-machi, Matsue, Shimane, 690-8506 Japan; 3 Department of Surgery, Masuda Red Cross Hospital, Otoyoshi-cho, Masuda, Shimane, 698-8501 Japan; 4 Department of Surgery, Unnan City Hospital, Daito-cho, Unnan, Shimane, 699-1221 Japan; 5 Department of Surgery, Izumo City General Medical Center, Nadabun-cho, Shimane, 691-0003 Japan

**Keywords:** Adjuvant chemotherapy, Esophagectomy, Neoadjuvant chemotherapy, S-1, Esophageal cancer

## Abstract

**Background:**

Despite advances in surgical techniques, long-term survival after esophagectomy for esophageal cancer remains unacceptably low, and more effective perioperative chemotherapy is expected. However, an important concern regarding the application of postoperative adjuvant chemotherapy is treatment toxicity. We aimed to evaluate the feasibility of adjuvant chemotherapy with S-1 in patients after esophagectomy.

**Methods:**

We investigated the tolerability of a 2-week administration followed by 1-week rest regimen of S1 as postoperative adjuvant therapy in 20 patients with esophageal squamous cell carcinoma who received neoadjuvant chemotherapy (NAC) and 22 patients who did not receive NAC during 2011–2020.

**Results:**

In the non-NAC group, the mean and median relative dose intensity (RDI) were 78.7% and 99.4%, respectively, and 11 patients (50%) had altered treatment schedules. The corresponding rates in the NAC group were 77.9% and 100%, respectively, and nine patients (45%) had altered treatment schedules, with no significant difference among the groups. Moreover, 17 patients (77.2%) in the non-NAC group and 16 patients (80.0%) in the NAC group continued S-1 treatment as planned for one year postoperatively, with no significant difference in the S-1 continuation rate (p = 0.500). Seventeen of 22 patients (77.3%) and 15 of 20 patients (75.0%) experienced several adverse events in the non-NAC and NAC groups, respectively. The frequency, severity, and type of adverse events were consistent among patients with and without NAC.

**Conclusions:**

S-1 could be safely and continuously administered as adjuvant chemotherapy for patients with esophageal cancer regardless of NAC. Long-term prognosis should be evaluated for S-1 to become the standard treatment after esophagectomy.

## Background

Several studies have shown that nearly half of the patients who undergo resection for esophageal cancer develop tumor recurrence and metastasis within the first postoperative year [1–3]. In this context, perioperative chemotherapy is increasingly attracting attention, and the development of more effective treatment regimens is urgently needed.

In Japan, JCOG9204 was a phase III trial to comparing the survival benefit between surgery alone and surgery plus postoperative adjuvant chemotherapy with two courses of cisplatin plus fluorouracil (CF) in patients who underwent curative esophagectomy. Their results demonstrated that adjuvant chemotherapy improved disease-free survival but did not show significant differences in overall survival (OS) when compared with surgery alone [4]. Subsequently, the JCOG9907 phase III trial aimed to compare survival following postoperative adjuvant chemotherapy with CF versus preoperative chemotherapy. Their results showed that preoperative chemotherapy significantly improved OS, but there was no significant difference among the groups in terms of progression-free survival [5].

While the clinical importance of preoperative adjuvant chemotherapy is generally accepted, the effectiveness of postoperative adjuvant therapy in patients who have already received preoperative adjuvant chemotherapy followed by esophagectomy has not been sufficiently proven [6, 7]. Because esophagectomy for esophageal cancer is highly invasive, and many patients experience postoperative complications that result in frailty for several postoperative weeks, adjuvant chemotherapy appears to be a high-risk intervention of uncertain therapeutic value. Moreover, the optimal adjuvant chemotherapy regimen for patients who have undergone resection of esophageal cancer following neoadjuvant chemotherapy (NAC) has not yet been established.

Preoperative clinical stage diagnosis has low accuracy for the prediction of the pathological stage, especially in resectable, localized esophageal cancer [8–10]. Preoperative chemotherapy or chemoradiotherapy is the standard treatment for node-positive, locally-advanced esophageal cancer; however, it may not be administered to patients with false-negative nodes or those with indications, because the preoperative clinical stage diagnosis and final pathological evaluation are occasionally discordant. Therefore, development of a feasible and effective adjuvant chemotherapy regimen is required for the improvement of long-term survival.

Given that previous clinical trials have proved the low tolerability of postoperative intravenous adjuvant chemotherapy, postoperative adjuvant therapy with an oral anticancer drug that does not burden the patient is desired. S-1, the only oral anticancer drug reimbursed for the treatment of esophageal cancer, has been proved to be tolerable in patients with gastric cancer and has been reported to improve long-term prognosis [11]. The present study aimed to retrospectively determine the tolerability and safety of adjuvant chemotherapy with S-1 in patients who underwent esophagectomy and received NAC in comparison with those who did not receive NAC.

## Patients and methods

### Patients

We retrospectively reviewed 53 consecutive patients who received postoperative adjuvant chemotherapy with S-1 after curative esophagectomy for histologically diagnosed esophageal squamous cell carcinoma (ESCC) between January 2011 and December 2020 at our institute. Inclusion criteria were patients with stage I, II, and III histologically confirmed ESCC after curative esophagectomy [12]. At our institution, preoperative chemotherapy or chemoradiotherapy (CRT) is performed for node-positive, locally advanced esophageal cancer in accordance with the Esophageal cancer practice guidelines 2017 edited by the Japan Esophageal Society [13, 14]. The standard NAC regimen is CF therapy; concurrent CRT with cisplatin plus 5-fluorouracil is provided for patients with suspected invasion to adjacent organs, and docetaxel, cisplatin plus 5-fluorouracil (DCF) therapy is provided for patients with suspected advanced lymph node metastasis. Curative resection was defined as complete tumor removal with microscopically negative resection margins. All patients underwent thoracoscopic subtotal esophagectomy with three-field lymph node dissection and reconstruction using the gastric conduit with anastomosis of the cervical esophagus and the gastric conduit.

The requirement for written informed consent was waived owing to the retrospective nature of this study. This study was approved by our institutional ethical review board (study number 20220422-2) and was conducted in accordance with the Declaration of Helsinki and the Japanese Ethical Guidelines for Clinical Studies.

### Postoperative adjuvant chemotherapy

Oral S-1 (tegafur, gimeracil, oteracil potassium; Taiho Pharmaceutical Co., Ltd, Tokyo, Japan) was administered twice daily for 2 weeks, followed by 1-week rest, as one course, within 10 weeks post-surgery. This 3-week cycle was repeated for up to 1 year after the start of oral administration [15]. Treatment was continued until unacceptable toxicity despite dose modification and temporary withdrawal of drug administration, patient’s refusal, or the treating physician’s decision.

The daily S-1 dose was calculated by body surface area (BSA). Patients with a BSA of < 1.25 m^2^ received 80 mg of S-1 daily, those with a BSA of 1.25–1.5 m^2^ received 100 mg daily, and those with a BSA of > 1.5 m^2^ received 120 mg daily.

The criteria for initiation of S-1 administration was as follows: (1) performance status of 0–1 on the Eastern Cooperative Oncology Group classification; (2) sufficient oral intake; (3) adequate organ function (white blood cell count ≥ 3,500 /mm^3^ and < 12,000 /mm^3^, neutrophil count ≥ 2,000 /mm^3^, hemoglobin level ≥ 9.0 g/dl, platelet count ≥ 100,000 /mm^3^, total bilirubin level ≤ 1.5 mg/dl, aspartate transaminase and alanine aminotransferase levels ≤ 100 IU/l; and (4) creatinine level ≤ 1.2 mg/dl and creatinine clearance > 60 ml/min in the Cockcroft-Gault equation.

Physical examination and biochemical analysis were performed at least every 3 weeks during the adjuvant chemotherapy. The adverse events were assessed and graded according to the National Cancer Institute Common Toxicity Criteria for Adverse Events, version 4.0 (https://ctep.cancer.gov/protocoldevelopment/electronic_applications/ctc.htm#ctc_40).

If patients experienced unacceptable hematological adverse events of grade 3 or higher, or non-hematologic adverse events of grade 2 or higher, S-1 treatment was temporarily discontinued until recovery to grade 2 or lower. When resuming treatment, the S-1 dose was reduced from 120 to 100 mg, from 100 to 80 mg, or from 80 to 50 mg per day.

### Evaluation of outcomes and statistical analysis

We compared the safety and feasibility of the administration of S-1 adjuvant chemotherapy for patients who underwent thoracoscopic esophagectomy with and without preoperative treatment. For feasibility evaluation, we calculated the continuation rate of S-1 oral administration for one year. The continuation rate was calculated by dividing the number of patients who received S-1 oral administration for one year by the total number of patients. The Kaplan–Meier method was used to calculate the continuation rate in patients who received postoperative S-1 adjuvant chemotherapy for one year, and differences were examined using the log-rank test. With the scheduled dosing period set to 12 months, the ratio of the actual cumulative dose to the planned cumulative dose was calculated to yield the relative dose intensity (RDI) [16]. Patients who had drug discontinuations for reasons other than adverse drug reactions (e.g., recurrence, death, surgery, or patient’s refusal) were deemed to be dropouts. Continuous variables and categorical variables were compared using the Mann–Whitney U test and chi-square test, respectively. A probability value of less than 0.05 was regarded as being statistically significant for all analyses. JMP software, version 16.0 (SAS Institute Inc., Cary, NC, USA), was used for performing all statistical analyses.

## Results

### Patient characteristics

**Fig. 1:**
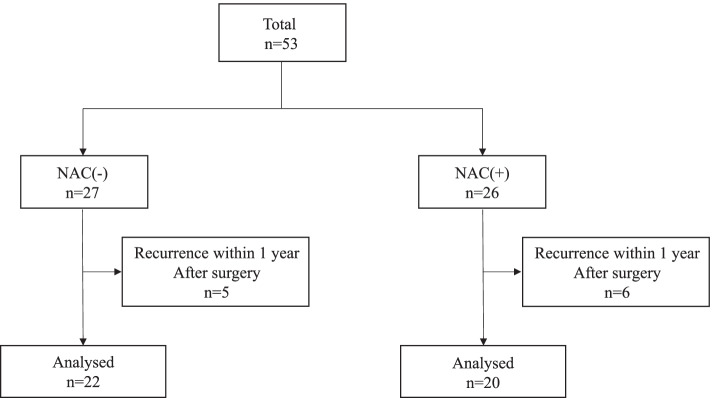
Consort diagram

Overall, 53 patients with ESCC received adjuvant chemotherapy with S-1 after curative esophagectomy. Twenty-six patients received NAC, and six patients were excluded from the analysis because of tumor recurrence within 1 year of surgery. Twenty-seven patients underwent upfront esophagectomy without preoperative treatment, and five patients were excluded from the analysis because of tumor recurrence within 1 year of surgery (Fig. 1). We analyzed 20 patients who received NAC treatment and 22 patients who did not receive NAC treatment without tumor recurrence for 1 year after surgery. There was no significant difference in patients ’ background characteristics among the two groups, and there was no difference in the occurrence of postoperative complications regardless of NAC (Table 1).

**Table 1:**
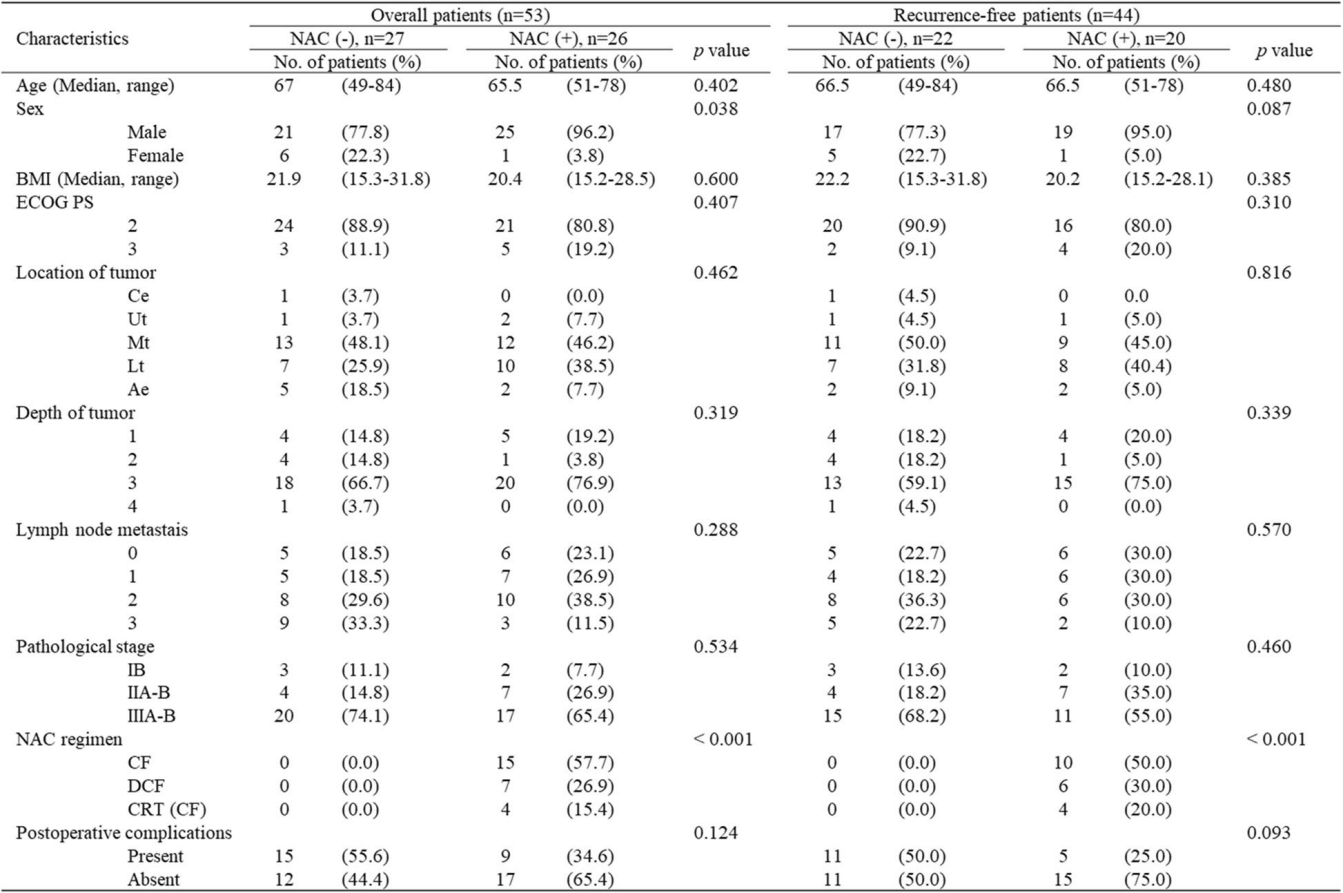
Patients’ characteristics

*NAC* Neoadjuvant chemotherapy, *BMI* Body mass index, *ECOG PS* Eastern Cooperative Oncology Group Performance Status, *Ce* Cervical esophagus, *Ut* Upper thoracic esophagus, *Mt* Middle thoracic esophagus, *Lt* Lower thoracic esophagus, *Ae* Abdominal esophagus, *CF* Cisplatin plus 5-Fluorouracil, *DCF* Docetaxel cisplatin plus 5-Fluorouracil, *CRT(CF)* Concurrent chemoradiotherapy with cisplatin plus 5-Fluorouracil

### Feasibility and tolerability of S-1

The treatment events are summarized in Table 2. The mean and median time from surgery to the initiation of S-1 administration in the non-NAC group were 83.8 and 74 days (interquartile range [IQR], 61.8–95.8 days), respectively. The corresponding values in the NAC group were 67.6 and 64 days (IQR, 51.3–81.8 days), respectively, with no significant differences in the time from surgery to the initiation of S-1 administration among the two groups (*p* = 0.110).

**Table 2:**
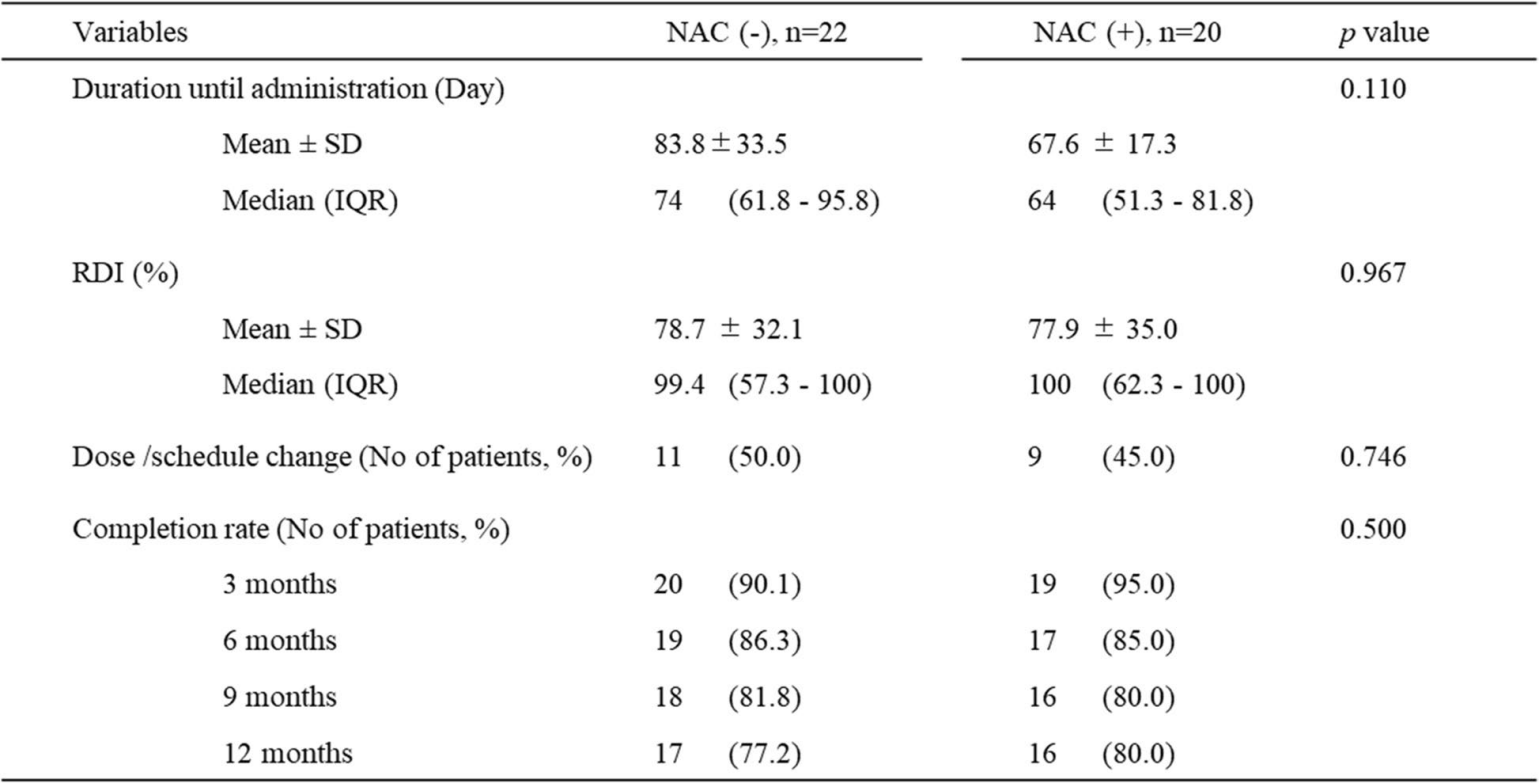
Feasibility and tolerability

*NAC* Neoadjuvant chemotherapy, *SD* Standard deviation, *IQR* Interquartile range, *RDI* Relative dose intensity

In the non-NAC group, the mean and median RDI values were 78.7% and 99.4% (IQR, 57.3–100), respectively, and 11 patients (50%) discontinued treatment, reduced doses, or changed treatment schedules. Similarly, in the NAC group, the mean and median RDI values were 77.9% and 100% (IQR, 62.3–100), respectively, and the treatment schedule was modified in nine patients (45%), with no significant difference among the two groups.

In the non-NAC group, the number of patients who continued S-1 treatment as planned for 1 year was 20 (90.1%), 19 (86.3%), 18 (81.8%), and 17 (77.2%) at 3, 6, 9, and 12 months after surgery, respectively. In the NAC group, 19 patients (95.0%) continued S-1 treatment for 3 months; 17 patients, for 6 months (85.0%); and 16 patients (80%), for 9 and 12 months. There was no significant difference in the S-1 continuation rate among the two groups (*p* = 0.500).

### Adverse events due to S-1

The adverse events due to S-1 are listed in Table 3. Seventeen of 22 patients (77.3%) and 15 of 20 patients (75.0%) experienced several adverse events in the non-NAC and NAC groups, respectively.

**Table 3:**
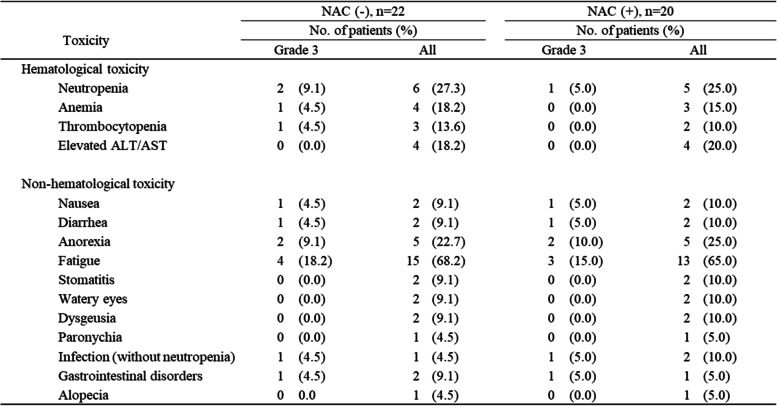
Adverse events

*NAC* Neoadjuvant chemotherapy, *ALT* Alanine aminotransferase, *AST* Aspartate aminotransferase

Grade 3 or higher hematological toxicities included neutropenia (9.1%), anemia (4.5%), and thrombocytopenia (4.5%), and most of the other adverse events were grade 2 or lower in the non-NAC group. Additionally, grade 3 or higher non-hematological toxicities included only neutropenia (5.0%), and most of the other adverse events were grade 2 or lower in the NAC group.

The most frequent grade 3 or higher non-hematological toxicity was fatigue (18.2% and 15.0% in the non-NAC and NAC groups, respectively). The frequency, severity, and type of adverse events were fairly consistent among patients with and without NAC, and the incidence of non-hematologic toxicity did not differ significantly among the two groups. The other frequent non-hematological toxicity was anorexia. Five patients (22.7%) developed anorexia and two patients (9.1%) had a grade 3 or higher toxicity in the non-NAC group. Five patients (25.0%) developed anorexia and two patients (10.0%) had grade 3 or higher toxicity in the NAC group. Patients with grade 3 toxicities were treated with an intravenous infusion at most thrice, but no patients required hospitalization, and all patients improved rapidly.

None of the patients had grade 4 toxicity, and there were no treatment-related deaths. All patients, whether or not they had hematological or non-hematological toxicity, had a manageable clinical condition with appropriate medical care.

### Treatment continuation rate based on postoperative complications

**Fig. 2:**
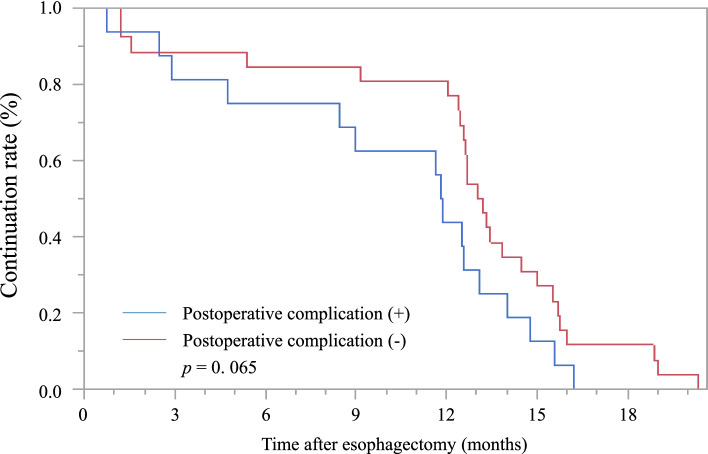
Comparison of the treatment continuation rates based on postoperative complications

The Kaplan–Meier method was used to compare the continuation rates of adjuvant chemotherapy based on postoperative complications. Although there was no significant difference, the S-1 continuation rate through oral administration tended to be lower in the group with postoperative complications (*p* = 0.07) (Fig. 2). Treatment discontinuation was more often observed during the first 3–4 months in both the groups.

## Discussion

The present study showed that S-1 therapy was safe as an adjuvant chemotherapy regimen for patients with esophageal cancer regardless of whether NAC was administered.

Esophagectomy plays a pivotal role in the treatment of esophageal cancer. Despite advances in surgical techniques, long-term survival after surgery for advanced esophageal cancer has remained unacceptable owing to poor survival. Hence, there is a need for more effective perioperative chemotherapy regimens [1, 2]. However, whether or not to add adjuvant chemotherapy to esophageal cancer surgery remains controversial because treatment toxicity is an important concern for the application of postoperative adjuvant chemotherapy [6, 7]. The main purpose of adjuvant chemotherapy is to eradicate micrometastatic tumor cells. Therefore, it is essential to continue chemotherapy with minimal duration and dose to ensure that these tumor cells are eradicated [17, 18]. A regimen involving 2-week S-1 administration followed by a 1-week rest (3-week regimen) was devised with an expectation to reduce toxicity and improve drug adherence while maintaining the same dose of S-1 as the standard 6-week regimen (4-week S-1 administration followed by a 2-week rest). This regimen was established based on the knowledge that the median time required for marrow suppression and the onset of non-hematological toxicity were 22 days and 15 days, respectively, and that a drug-free interval of 3 weeks after the start of S-1 treatment may reduce the incidence of adverse events. Previous reports of patients with gastric cancer have reported that a 3-week regimen improves medication adherence and reduces adverse events while maintaining the same dose of S-1 as the standard 6-week regimen [19, 20]. Based on the above findings, we provided a 3-week regimen of postoperative adjuvant therapy with S-1 for patients after surgery for advanced esophageal cancer at our institution.

The treatment completion rate of S1 was as high as 80% in the NAC-treated group and 80% in the NAC-non-treated group, which was almost the same as the result of the Adjuvant Chemotherapy Trial of S-1 for Gastric Cancer (ACTS-GC), which examined the efficacy of adjuvant therapy with S1 in patients with gastric cancer [11]. The quality of life of patients is affected more by non-hematological toxicity such as loss of appetite, malaise, and nausea than by hematological toxicity [21]. In this study, S-1 treatment could be continued owing to the low incidence of grade 1–2 non-hematological toxicity. In addition, according to ACTS-GC studies, the incidence of grade 3/4 neutropenia was approximately 5%. In this study, grade 3 neutropenia was observed equally in 18% and 18% of patients in the NAC-treated and non-NAC-treated groups, respectively, and was transient and manageable. To the best of our knowledge, this is the first study to demonstrate that the feasibility and toxicity profile of adjuvant chemotherapy with S-1 was similar in patients who received and did not receive NAC.

The 3-week regimen was considered equally acceptable in terms of RDI, completion rate, and frequency of adverse events, demonstrating better feasibility in both groups. Our results suggested that S-1 adjuvant chemotherapy was safe and feasible for patients with esophageal cancer who underwent esophagectomy regardless of NAC administration. Therefore, it is unnecessary to avoid chemotherapy or to reduce the dose of S-1 in patients who received NAC. If future studies reveal that the 3-week regimen has a detectable preventive effect on long-term prognosis, then this regimen will be established as a safe postoperative adjuvant chemotherapy regimen.

Although detailed data on the nutritional status of patients were not evaluated in this study, several studies have shown a significant reduction in nutrition-related parameters, such as the obesity index, serum albumin, and hemoglobin in patients with esophageal cancer [22, 23]. Chemotherapy can cause both deterioration of nutritional status due to toxicity and, conversely, improvement of nutritional status due to tumor shrinkage. Therefore, it is difficult to determine whether nutritional status changes are due to chemotherapy or the detrimental effects of surgery. For patients with gastric cancer, a decrease in nutritional indicators such as body weight and lean body mass has been reported to reduce compliance with anticancer drug treatment [24, 25]. Thus, maintaining the nutritional status during adjuvant chemotherapy is important, given that drug compliance can lead to improved survival. A decline in health-related quality of life may last from 6 months to 3 years until after the surgery [26]. Fortunately, in this study, only two patients (9.1%) experienced grade 3 or higher toxicity in the non-NAC group, and only two patients (10.0%) experienced grade 3 or higher toxicity in the NAC group. In addition, S-1 may not have had a serious impact on nutritional status (due to adverse events), as most patients did not develop severe anorexia requiring discontinuation of adjuvant chemotherapy. Detailed nutritional assessments and studies of perioperative nutritional interventions during adjuvant therapy in patients undergoing esophagectomy are required.

Esophagectomy is a highly invasive surgery requiring three-field lymph node dissection and reconstruction with a gastric conduit, despite advances in minimally invasive esophagectomy [27, 28]. Therefore, during the early postoperative period, patients would have not yet recovered from surgical stress and are more likely to experience adverse events such as loss of appetite and nausea. To resolve these issues, it is necessary to determine the appropriate initiation time and criteria for adjuvant therapy, as well as the factors that are likely to cause adverse events.

The present study has some limitations, which must be considered when interpreting the results. First, this was a retrospective single-center study with a small sample size, which may account for the lack of meaningful statistical data. Thus, larger sample sizes are needed to draw well-founded conclusions. However, because the toxicity profile in this study was recorded using a check sheet in most patients at the time of consultation during S-1 adjuvant treatment, sufficient information on adverse events was collected, and the results of this study can be reliable. Second, the patients’ follow-up period was too short to assess the therapeutic effect of the S-1 adjuvant therapy on long-term outcomes. The main purpose of the present study was to assess the toxicity profile, not the efficacy of the adjuvant S-1 treatment. Long-term survival outcomes are a topic for future research, and our data support the administration of adjuvant chemotherapy for patients who have undergone curative esophagectomy after preoperative chemotherapy. Third, selection bias was likely among patients treated with S-1. Although there was no significant difference, patients who received NAC were younger, had fewer postoperative complications, and the time from surgery to the initiation of S-1 administration was shorter than that in the non-NAC group. Among patients of the same stage, some received postoperative adjuvant chemotherapy and some did not. Even the first-stage patients received postoperative adjuvant therapy on request, resulting in a potential selection bias. Patients with esophageal cancer often have comorbidities such as chronic obstructive pulmonary disease and liver dysfunction as well as physiological problems that can cause greater drug toxicity than that in patients with other cancers [29, 30]. Therefore, it is important to determine the clinical predictors of serious adverse events and early discontinuation of S-1 adjuvant therapy.

In conclusion, S-1 could be safely and continuously administered as adjuvant chemotherapy in patients with esophageal cancer regardless of the administration of NAC. Although S-1 has a very high antitumor effect on many carcinomas, its long-term prognosis needs to be evaluated in a prospective study to become the standard treatment for postoperative patients with esophageal cancer. Furthermore, to improve the therapeutic outcomes, it is also important to develop individual treatment strategies in the future.

## Data Availability

The datasets obtained and/or analyzed during the current study are available from the corresponding author on reasonable request.

## Abbreviations

ACTS-GC: Adjuvant Chemotherapy Trial of S-1 for Gastric Cancer
Ae: Abdominal esophagus
BMI: Body mass index
BSA: Body surface area
Ce: Cervical esophagus
CF: Cisplatin plus 5-Fluorouracil
ESCC: Esophageal squamous cell carcinoma
IQR: Interquartile range
Lt: Lower thoracic esophagus
Mt: Middle thoracic esophagus
NAC: Neoadjuvant chemotherapy
OS: Overall survival
RDI: Relative dose intensity
SD: Standard deviation
Ut: Upper thoracic esophagus
